# Conservative Management of Cervical Pregnancy with the Administration of Methotrexate and Potassium Chloride: A Case Report

**DOI:** 10.1155/2022/1352868

**Published:** 2022-11-07

**Authors:** Mojgan Javedani Masroor, Anna Zarei, Hossein Sheibani

**Affiliations:** ^1^Shahid Akbar Abadi Clinical Research Development Unit, School of Medicine, Iran University of Medical Sciences, Tehran, Iran; ^2^Clinical Research Development Unit, Imam Hossein Hospital, Shahroud University of Medical Sciences, Shahroud, Iran

## Abstract

*Background. C*ervical pregnancy is a rare form of ectopic pregnancy in which the fetus implants and grows inside the endocervical canal. This report aims at introducing a case of successful conservative management of cervical ectopic pregnancy. *Case presentation.* The patient was a 35-year-old woman, who had received treatment for primary infertility for 5 years. She complained of painless bleeding on day 37 of gestational age with a start point from 10 days before. The patient had stable vital signs and was referred to Shahid Akbar Abadi Hospital in Tehran affiliated with the Iran University of Medical Sciences. In the ultrasonography, the pregnancy sac and the yolk sac with the embryonic pole with a positive fetal heart rate were presented and located near the internal os, so the cervical pregnancy was diagnosed, and after treatment with intramuscular methotrexate and intra-amniotic administration of potassium chloride, a gradual decrease in *β*-HCG levels was observed without the need for additional interventional treatment. *Conclusion.* The primary takeaway of our report is that the conservative treatment, including intramuscular methotrexate and intrauterine potassium chloride administration, may be effective in treating cervical pregnancy that can be detected early without the use of curettage.

## 1. Background

Cervical pregnancy (CP) is a rare form of ectopic pregnancy (EP) in which the fetus implants and grows inside the endocervical canal. Nontubular EPs make up less than 1% of all EPs, although their occurrence has increased in recent years [1, 2]. The incidence of CP varies between one in 1000 and one in 18000 pregnancies and 1% of all EPs [2].

Although the cause of CP is unknown, it has increased in recent years due to the increasing use of assisted reproductive techniques (in vitro fertilization). Other predisposing factors include previous cesarean section delivery, cervical surgery, previous dilatation and curettage, smoking, and the use of intrauterine devices [3].

Patients present with painless vaginal bleeding, but there have been reports of pain and cramps. Clinical diagnostic criteria for the diagnosis of cervical pregnancy are shown in Table 1 [4–6].

Ultrasound has been described as helpful in differentiating a true cervical pregnancy from developing spontaneous abortion. In such cases, magnetic resonance imaging (MRI) of the pelvis can be used as a compliment when the diagnosis is unclear. Ultrasound criteria for diagnosing cervical pregnancy are presented in Table 2 [5, 7].

Clinical diagnosis of CP is difficult. Other items that could be included in the list of differential diagnoses of cervical pregnancy include cervical carcinoma, cervical leiomyoma or protruding leiomyoma, trophoblastic tumor, and placenta previa [7].

If the diagnosis is delayed, severe vaginal bleeding and acute abdominal/pelvic pain may occur. Diagnosis often occurs late and due to massive blood loss in 50% of cases, an emergency hysterectomy is required [6]. Recently, the recommended protocol for the treatment of cervical ectopic pregnancy is fertility preservation rather than invasive surgery and hysterectomy [7].

Treatment for cervical ectopic pregnancies includes medical treatment with methotrexate and surgical treatment. The best treatment regimen for medical treatment has not yet been determined, but evidence suggests that single-dose or multidose regimens of methotrexate have been successful. In the cases of advanced pregnancies, especially if associated with fetal heart activity, the combined use of multiple doses of methotrexate (MTX) and potassium chloride (KCl) injections into the fetus or the amniotic fluid might be used. However, these injections must be done by a well-specialized person to avoid rupturing the membranes during the procedure [8]. Methotrexate is a folic acid analog that has been used successfully for the treatment of ectopic pregnancy ad blocks trophoblast cell division and prevents trophoblast proliferation [9].

In the current study, we described a case of cervical pregnancy that was managed with the intramuscular (IM) administration of methotrexate and intra-amniotic injection of KCl without the need for additional interventional treatment. This report aims at introducing a case of successful conservative management of cervical ectopic pregnancy.

## 2. Case Presentation

The patient was a 35-year-old woman, who had received treatment for primary infertility for 5 years. She had menarche at the age of 17, and her menstrual cycles were regular. The patient had a follicle-stimulating hormone (FSH) level of 1.613 (IU/L) and a luteinizing hormone (LH) level of 0.6 (IU/L) and was treated with letrozole over five days from the third to the seventh day of the menstrual cycle and then became pregnant.

She complained of painless bleeding on day 37 of gestational age with a start point from 10 days before. The patient had stable vital signs and was referred to Shahid Akbar Abadi Hospital in Tehran affiliated with the Iran University of Medical Sciences. Beta human chorionic gonadotropin (BHCG) was checked and was 6000 IU/l.

In physical examination, the abdomen was soft without distension and tenderness. Speculum examination was done slowly. Bleeding was less than that of the menstruation, and the cervix was completely closed. Hegar's sign and Chadwick's sign were both positive.

**Figure 1:**
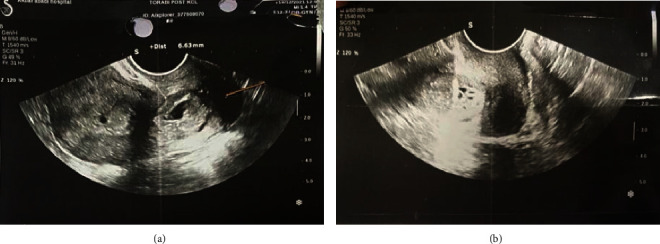
The obstetric ultrasonography shows the pregnancy sac and the yolk sac with the embryonic pole located near the internal os.

In the transvaginal ultrasonography, the pregnancy sac and the yolk sac with the embryonic pole with a positive fetal heart rate (FHR) were presented and located near the internal os, at the beginning of the cervical canal. The crown-rump length (CRL) was 4 mm, and the patient had normal endometrial thickness. Most of the pregnancy sac was seen in the proximal cervix, and there was a small part in the lower segment. The pregnancy bag had more blood than the other areas. There was a mild decidual reaction around the amniotic sac. Therefore, a cervical pregnancy was diagnosed, and the patient was hospitalized for further treatment (Figure 1).

Due to the stable vital signs, clinical condition of the patient, and the presence of embryonic echo, treatment with intramuscular MTX was selected. Potential risks and alternative therapies were explained to the patient, and informed written consent was obtained. Treatment consisted of intramuscular injection of MTX 5 mg/kg and folic acid 0.1 mg/kg alternately every other day for 4 days.

**Figure 2:**
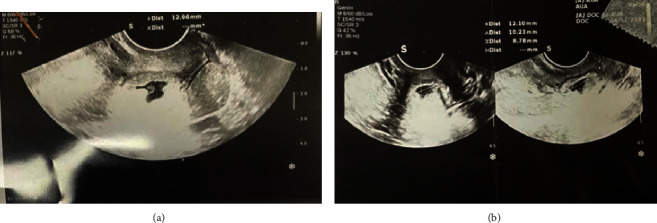
The 2^nd^ ultrasonography, a fetal echo (with FHR +) in the pregnancy bag.

**Figure 3:**
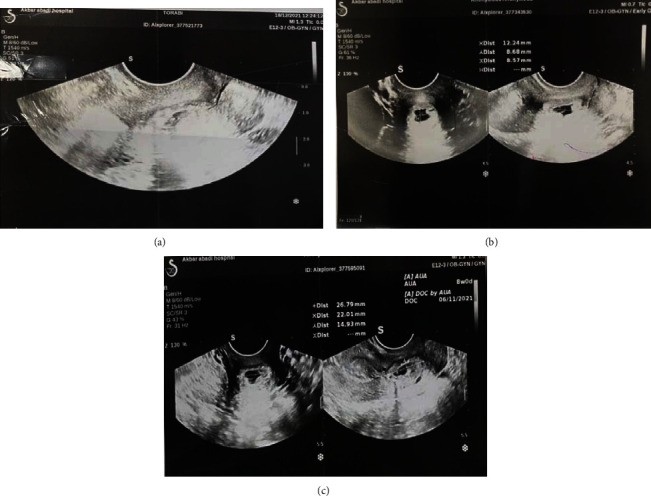
The 3^rd^ ultrasonography, a fetal echo (with FHR +) in the pregnancy bag.

Serial ultrasonography was done, and there was a fetal echo (with FHR +) in the pregnancy bag on days 7 and 14 (Figures 2 and 3), so one cc KCL was injected into the pregnancy bag under the guidance of ultrasound.

**Figure 4:**
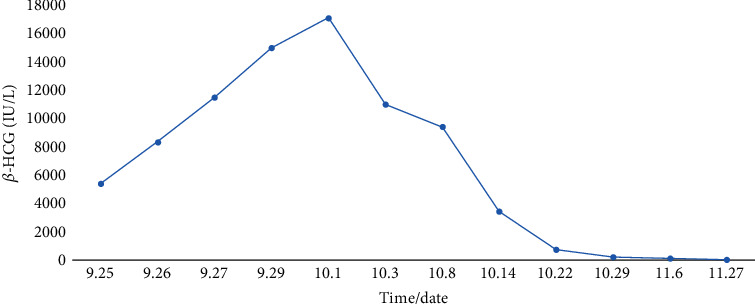
Changes in *β*-HCG levels during treatment.

After treatment with 4 doses of MTX, a gradual decrease in *β*-HCG levels was observed (Figure 4). The patient reported minimal vaginal discharge, with no pain or other symptoms, so she was discharged after 14 days with the recommendation of weekly follow up until the *β*-HCG levels decreased under the 10 IU/L.

Serial ultrasonography was done. As in the 3^rd^ ultrasonography, there was a fetal echo (with FHR +) in the pregnancy bag (Figure 3).

## 3. Discussion

Cervical pregnancies are rare in naturally conceived pregnancies and even after assisted reproductive technology. One of the differential diagnoses of cervical pregnancies is Cesarean scar pregnancies (CSPs). Cesarean scar pregnancies are more commonly seen today, both in the East and the West, as a result of a significant increase in the proportion of Cesarean deliveries over the last three decades [10].

The exact mechanism of ectopic pregnancy is unknown. The rapid migration of fertilized eggs through the uterus and changes in the ability of the endometrial lining to accept implantation and damage to the endometrial canal may all be the causative factors [11]. In the current study, in the past medical history, the patient was only treated with an ovulation-stimulating drug and did not receive intrauterine insemination (IUI) or in vitro fertilization (IVF).

The cause of pregnancy after IVF is not well understood. It is believed that it is related to the increase in contractions of the uterine gap junction in the luteal phase. This increase in contractions is due to an increase in progesterone [12, 13]. Authors such as Bennett et al. have accepted this hypothesis but continued to point out that women undergoing assisted reproductive techniques often have other risk factors for cervical pregnancy [14]. In the current study, there were no other risk factors for cervical pregnancy. However, the prevalence of cervical ectopic pregnancy has increased with assisted reproductive technologies [3]. It should be noted that cervical pregnancy is very dangerous if not considered correctly. If left untreated, it can eventually lead to a hysterectomy. However, in most women, the risk factor is unknown [15].

The ultrasonographic diagnosis of cervical pregnancy is to find the trophoblastic invasion of the cervix below the internal os [12]. In the current study, cervical ectopic pregnancy was diagnosed early based on the transvaginal and ultrasound examination of the patient.

In a study done by Vela et al., twelve cases of cervical pregnancy with the early diagnosis were evaluated, of whom none resulted in a hysterectomy, so the earlier diagnosis the less likely complications are [16].

We successfully treated one case of cervical pregnancy with an intramuscular injection of MTX combined with an intra-amniotic injection of potassium chloride without the need for further treatment with curettage and additional interventions.

The presence of a large number of management options highlights the fact that there is no standardized protocol, or we could say that no agreement has yet been reached on the optimal treatment for cervical pregnancy and CSP management. Several options have been used over the last three decades for the conservative nonoperative management of cervical pregnancy [10].

However, there are scientific limitations due to the rarity of cases; the trend of modern clinical practice is more towards conservative nonoperative management [12, 17]. Management of ectopic pregnancy depends on several factors including gestational age, fetal heart activity, patient stability, interest in maintaining fertility, and availability of medical and specialized facilities. Conservative nonoperative treatment includes administration of methotrexate in case of advanced gestational age and presence of FHR. Failure in conservative management, severe bleeding, and unstable hemodynamics requires surgical intervention including curettage with a Foley catheter for uterine tamponade, topical injection of prostaglandins, uterine artery embolization, bilateral ligation of the uterine or iliac artery, and hysterectomy for patients who are no longer interested in pregnancy [18, 19]. According to operative management, laparoscopic surgery and suturing is the single most effective treatment of CSP. Optimal repair of the uterine defect is essential for the prevention of another CSP [10].

In a study done by Samal et al. in 2015, a successful case of conservative treatment in a 29-year-old woman with GA = 7 who was treated with a combination of methotrexate and intra-amniotic injection KCL and curettage suction was presented [20]. A study done by Kadija et al. in 2016 showed efficient fertility-sparing conservative treatment, dilatation, and curettage, of a 13-week cervical pregnancy followed by systemic methotrexate [21].

Hamouda et al. discussed the relative effectiveness and positive efficacy of intramuscular and single-dose MTX administration without the need for curettage in cervical pregnancy [22]. In a study done by Petousis et al. in 2015, a cervical pregnancy was successfully treated with the intramuscular injection of methotrexate (MTX) and intraamniotic administration of potassium chloride in a 41-year-old woman with a gestational age of 54 days.

According to the administration of an intra-amniotic feticide using potassium chloride (KCL), it may be effective in the treatment of cervical pregnancy without the need for performing curettage. Indeed, the effectiveness of exclusive administration of MTX has been reported to be as high as 81.3%, while the percentage is increased to 90% when MTX is combined with additional conservative methods [16].

Our patient was ideal for conservative management. However, the most important point in treating cervical pregnancy with MTX is patient selection and identification. These patients should be hemodynamically stable; the ectopic pregnancy should not be ruptured; there should be no complaints of severe bleeding and severe pelvic pain; the CRL size should not exceed 3.5_3 cm; the BHCG level should be less than 5000 IU/L, and the patient should be willing to participate in the next follow-up visits [23–25]. In our patient, the length of CRL was 4 mm; the maximum BHCG level was 160000 IU/L, and vital signs were stable. After intraamniotic administration of the KCL, a decrease in BHCG level was seen.

The effectiveness of the administration of MTX for cervical pregnancy has been reported to be as high as 81.3%. without the need for curettage, while a combination of systemic MTX along with additional conservative methods has a better prognosis and was found to be effective in 91% of cases [26].

## 4. Conclusion

The primary takeaway of our report is that the conservative treatment, including IM MTX and intrauterine KCL administration, may be effective in treating cervical pregnancy that can be detected early without the use of curettage.

## 5. Recommendation

Further multicenter observational or even randomized studies should be done to evaluate the relative effectiveness of various therapeutic protocols and also assess the issue of cost-effectiveness of invasive vs. conservative management.

## Figures and Tables

**Table 1:** Clinical diagnostic criteria for the diagnosis of the cervical pregnancy.

1	Painless bleeding from the uterus after a period of amenorrhea
2	The uterus is smaller than the enlarged cervix, which dilates and surrounds it.
3	The internal os may not be dilated. The closed internal os with partially open external os.
4	Bulky cervical speculum with bluish mucosa. The ovular tissue is completely placed in the endocervix
5	Severe bleeding due to cervical manipulation.

**Table 2:** Ultrasound criteria for diagnosing cervical pregnancy.

1	Observation of pregnancy sac in the cervix
2	Heart movement should be detected below the surface of the internal opening.
3	Absence of intrauterine pregnancy
4	Observing the uterus in the form of an hourglass with an enlarged cervix
5	No movement of the pregnancy sac due to the pressure of the vaginal probe (i.e., no sliding sign, which is usually seen in incomplete abortions.

## Data Availability

The datasets used and/or analyzed during the current study are available from the corresponding author on reasonable request.

## Abbreviations

(BHCG): Beta human chorionic gonadotropin
(CP): Cervical pregnancy
(EP): Ectopic pregnancy
(FHR): Fetal heart rate
(FSH): Follicle-stimulating hormone
(IM): Intramuscular
(IUI): Intrauterine insemination
(IVF): In vitro fertilization
(LH): Luteinizing hormone
(MRI): Magnetic resonance imaging
(MTX): Methotrexate.
